# Weight Loss in Advanced Cancer: Sex Differences in Health-Related Quality of Life and Body Image

**DOI:** 10.3390/life12010105

**Published:** 2022-01-12

**Authors:** Charlotte Goodrose-Flores, Helén Eke, Stephanie E. Bonn, Linda Björkhem-Bergman, Ylva Trolle Lagerros

**Affiliations:** 1 Department of Medicine (Solna), Clinical Epidemiology Division, Karolinska Institutet, SE-171 64 Solna, Sweden; helen.eke@regionstockholm.se (H.E.); stephanie.bonn@ki.se (S.E.B.); ylva.trolle@ki.se (Y.T.L.); 2 Center of Obesity, Academic Specialist Center, Stockholm Health Services, SE-113 64 Stockholm, Sweden; 3 Department of Neurobiology, Care Sciences and Society, Division of Geriatrics (Huddinge), Karolinska Institutet, SE-141 83 Huddinge, Sweden; linda.bjorkhem-bergman@ki.se; 4 Stockholms Sjukhem, Palliative Medicine, SE-112 19 Stockholm, Sweden

**Keywords:** cancer, palliative, weight loss, health-related quality of life, body image, sex differences

## Abstract

Weight maintenance is a priority in cancer care, but weight loss is common and a serious concern. This study explores if there are sex differences in the perception of weight loss and its association to health-related quality of life (HRQoL) and body image. Cancer patients admitted to Advanced Medical Home Care were recruited to answer a questionnaire, including characteristics, the HRQoL-questionnaire RAND-36, and a short form of the Body Image Scale. Linear regression analyses stratified by sex and adjusted for age were performed to examine associations between percent weight loss and separate domains of HRQoL and body image score in men and women separately. In total, 99 participants were enrolled, of which 80 had lost weight since diagnosis. In men, an inverse association between weight loss and the HRQoL-domain physical functioning, β = −1.34 (95%CI: −2.44, −0.24), and a positive association with body image distress, β = 0.22 (95%CI: 0.07, 0.37), were found. In women, weight loss was associated with improvement in the HRQoL-domain role limitations due to physical health, β = 2.02 (95%CI: 0.63, 3.41). Following a cancer diagnosis, men appear to experience weight loss more negatively than women do. Recognizing different perceptions of weight loss may be of importance in clinical practice.

## 1. Introduction

Malnutrition and weight loss are serious consequences following a cancer diagnosis and its treatment [1] and are commonly found already at the first oncology-related visit [2]. Interventions to treat the condition can lead to improved treatment outcomes [3]. Diagnostic criteria for malnutrition include percent weight loss, low body mass index (BMI, kg/m^2^), reduced food intake or assimilation, inflammation, and reduced muscle mass [4]. While muscle loss in advanced cancer is more prevalent in men than in women [5,6], a change in body composition with involuntary muscle loss has been shown to be critical for quality of life (QoL) and mortality [7,8].

Percent weight loss is an independent predictor of survival in patients diagnosed with cancer [9]. Therefore, weight maintenance is a priority. However, weight loss is challenging to treat in this population and often requires both volitional and artificial nutrition interventions [10,11,12]. For this reason, dietary interventions are advised to be initiated early [13], as this improves the chances to prevent weight loss [14].

There are many factors driving weight loss in patients diagnosed with cancer, including inadequate energy intake, often related to altered taste and smell, and metabolic changes [13]. In simple starvation, lipolysis is upregulated to meet energy needs, which is a metabolic response to preserve muscle mass. Such protective pathways are lacking in individuals with cancer, and hence, muscle mass is wasted [10]. In overweight and obese patients, significant weight loss might go unnoticed and not be considered as alarming, even though the depletion of muscle mass might be severe [15].

Weight loss has been associated with a low QoL already at the beginning of the cancer treatment [16], and it is associated with a demand for nutritional counseling [14]. Wellbeing has been shown to decrease with weight loss in all stages of cancer cachexia [17].

Hence, QoL and wellbeing are important factors to consider in relation to weight loss in patients diagnosed with cancer.

Body image is a multidimensional construct that includes the individual’s perception of, and attitude toward their body [18]. In patients diagnosed with cancer, body image is an important factor when evaluating QoL, as the body may change due to the disease, treatment, or both [19]. The possibly permanent changes to body appearance might therefore significantly affect body image. However, the link between weight loss in patients diagnosed with cancer and health-related quality of life (HRQoL), body image, and sex is still to be explored. 

The aim of this study was to advance the understanding of the perception of weight loss following a cancer diagnosis, by examining how it relates to HRQoL and body image, and whether there are sex differences. This understanding could increase the possibilities for the clinician to intervene with targeted nutrition counseling. To our knowledge, this is the first study that explores weight loss in a palliative cancer population from this perspective.

## 2. Materials and Methods

### 2.1. Study Design and Populations

This study was conducted between March of 2018 and February of 2020, among patients admitted to Advanced Medical Home Care (ASIH), Stockholm Södra, Sweden. Details about ASIH can be found elsewhere [20]. Most patients enrolled in ASIH suffer from a chronic, life-threatening disease in a palliative phase [20]. The median enrollment time is 3 to 4 months, ranging from days to years [20].

In total, 178 patients, diagnosed with cancer, were given oral information about the study by a dietitian. Thereafter, they were sent written information about the study, an informed consent to sign, the study questionnaire, and a pre-paid return envelope. The inclusion criteria for the study were: ≥18 years old, diagnosed with advanced cancer, sufficient knowledge in Swedish to understand and answer the questionnaire, and signed informed consent. Both men and women were included in the study.

### 2.2. Patient Demographics

A questionnaire was used to collect demographic information, including age, level of education, marital status, cancer type, comorbidities, and smoking status.

### 2.3. Anthropometrics

Patients were asked about height, as well as pre-diagnostic and present weight. The pre-diagnostic weight was defined as the weight prior to diagnosis, and the present weight was the weight at the study inclusion. Weights were verified by checking the participants’ medical records. The pre-diagnostic and the present weights were used to calculate percent weight change and BMI.

### 2.4. Health-Related Quality of Life

RAND-36 [21] consists of 36 questions and was used to assess HRQoL within eight health domains: physical functioning, role limitations due to physical health, role limitations due to emotional problems, energy/fatigue, emotional well-being, social functioning, pain, and general health. Scores ranged from 0 to 100, where a higher score indicated a more favorable health state.

### 2.5. Body Image

The Body Image Scale assessed body image distress and has been developed for use in cancer patients that might experience body concerns [19]. The 10-item questionnaire was translated into Swedish by our research team and a short form was created by removing three items that has been reported as frequently missing [19]. We followed the original scoring model with four possible responses computing a maximum summary score of 21. The responses ranged from “not at all” (0 points) to “very much” (3 points), where a higher score indicated a more distressed body image.

In the analysis of both HRQoL and body image, missing answers were replaced by the imputed average score of the other questions. At least 50% of the questions had to be answered to obtain a score.

### 2.6. Self-Measured Health 

The participants’ self-measured health was assessed using a visual analogue scale (VAS) [22]. The participants were instructed to rate their current health on a scale ranging from 0 to 100, where a higher score indicated better health.

### 2.7. Data Analysis

Descriptive statistics are presented as mean (SD) or n (%). The cohort was stratified by sex and compared using independent t-tests and chi2-tests for continuous and categorical variables, respectively. Linear regression was used to examine the association between percent weight loss and each HRQoL-domain and body image score, stratified by sex. Since the aim was to examine how weight loss was associated with HRQoL and body image, only participants that had lost weight after diagnosis were included in the analyses. The linear regression models were adjusted for age. Age was selected based on the assumption that it affected both body image and HRQoL [23]. Sensitivity analysis adjusting for additional covariates, including level of education, previously attempted weight loss prior to diagnosis, and having a BMI ≥ 25 kg/m^2^ prior to diagnosis, were also performed. Each regression analysis was repeated after exclusion of outliers among the residuals.

In further analysis of body image, independent t-tests were applied to examine if body image score differed depending on if there was a history of ever having attempted weight loss prior to diagnosis or not, and if weight had been lost since the cancer diagnosis or not. Finally, Spearman’s rank test was applied to examine the correlation between body image score and the health scale, stratified by sex, and repeated including only participants that had lost weight after diagnosis.

*P*-values < 0.5 were considered statistically significant. Statistical analyses were performed using Stata 14 (Stata Corporation, College Station, TX, USA).

## 3. Results

### 3.1. Study Population

In total, 99 participants signed the informed consent and returned the questionnaire. Of these, 56% were men. Most of the participants had lost weight since diagnosis (n = 80), although some had not (n = 15). The pre-diagnosis and/or the present weights were missing for four participants. Among those who had lost weight since diagnosis, the average weight loss was 11% of the pre-diagnosis body weight. There were no differences regarding age, level of education, marital status, smoking, or comorbidities between men and women. However, more women (n = 21) than men (n = 12) had attempted to lose weight prior to diagnosis (*p* = 0.009). General and clinical characteristics are summarized in Table 1. The different cancer types in the study cohort are presented in Table 2.

### 3.2. Heath-Related Quality of Life

There were no significant differences in scores of the eight HRQoL-domains between men and women (Table 1). Results from the linear regression models are presented in Table 3. An inverse association was found between weight loss and physical functioning β = −1.34 (95%CI:−2.44, −0.24) in men. In women, there was an association between weight loss and improvement in the domain role limitations due to physical health β = 2.02 (95%CI: 0.63, 3.41). These results remained significant in sensitivity analyses. There were no other significant associations between weight loss and any HRQoL-domain. The results remained significant in sensitivity analysis of multivariable adjusted models (data not shown).

### 3.3. Body Image

Women reported a significantly more distressed body image than men (*p* = 0.02). This remained significant also when only participants that had lost weight since diagnosis were included in the analysis. As presented in Table 3, there was an association between weight loss and body image distress, β = 0.22 (95%CI: 0.07, 0.37), in men. This was not seen in women. These results remained significant in sensitivity analyses.

Further analyses of body image showed more distress among participants who had attempted to lose weight prior to diagnosis compared to those who had not (*p* = 0.046). However, body image score did not differ depending on whether weight was lost since diagnosis or not (*p* = 0.38). Among women, body image distress was inversely associated with self-measured health among those who had lost weight since diagnosis (r = 0.48, *p* = 0.008). This was not seen in men.

## 4. Discussion

This study indicates that there are sex differences in the perception of weight loss following a cancer diagnosis. In men, weight loss was inversely associated with physical functioning and associated with body image distress. In contrast, weight loss in women was associated with improvement in the HRQoL-domain role of limitations due to physical health. Among the entire study population, greater body image distress was seen in those who had attempted to lose weight prior to diagnosis, compared to those who had never made such an attempt. Among those who had lost weight since diagnosis, body image score was inversely associated with self-measured health in women, but not in men.

Females in our study reported a more distressed body image than men. In line with our results, a Finnish study in adolescent female leukemia survivors found that their female population reported an impaired body image, compared to healthy controls [24]. Further, we found that weight loss was associated with an improvement in the HRQoL-domain role limitations due to physical health in women. The results from this study might be interpreted as such that women have a positive perception of weight loss, even though it is the consequence of a serious medical condition known to increase mortality [9]. Hence, women experiencing a serious illness might continue relating their bodies to sociocultural ideals, whereby female bodies with a BMI of 18 to 19 kg/m^2^ are rated as more attractive [25,26]. Men experienced a decline in physical functioning with weight loss, which may be interpreted as an unwanted distancing from traditionally masculine traits such as strength [27].

### Strengths and Limitations

This study has both strengths and limitations. In addition to the nature of a palliative cohort, limitations also include the fact that the study is based on a small convenience sample, and that the data were retrieved at just one time point using self-reported questionnaires. Further, we did not collect, and therefore did not adjust for, information about cancer treatment phase or concurrent depression. The cancer disease was assumed to be the major cause of weight loss in the patients included in the study, although other conditions could have had an impact on HRQoL [28], body image distress [29], and the ability to comply with dietary recommendations [11,29]. It may be a limitation that we did not ask the study participants what they themselves thought was the reason for weight loss. However, mental health is carefully screened for on a weekly basis, following a local protocol, in all patients enrolled at Advanced Medical Home Care, and thoroughly treated with both pharmacological and non-pharmacological interventions. Furthermore, we did not collect data on nutritional variables other than BMI, nor did we collect data on physical activity, although most of the patients were assessed as having very limited ability to conduct any physical activity. However, the sex difference in the perception of weight loss in advanced cancer, regardless of cause, is interesting since weight loss is strongly associated with poorer prognosis in these patients [9].

Applying a personalized gender perspective in nutrition counseling might add the necessary attention and understanding to underpin patients’ compliance to individual nutritional goals. Therefore, despite the limitations, our results may still be of value in clinical practice and benefit any health care professional caring for cancer patients.

This is, to the best of our knowledge, the first study to report sex differences in the perception of weight loss in patients diagnosed with advanced cancer. Another strength is that we also verified weights by their medical records, and we have used questionnaires that are well established within the research community [19,21,22].

## 5. Conclusions

This study suggests that sex may affect the perception of weight loss following a cancer diagnosis, as women appear to experience weight loss more positively than men. To assess the perception of weight loss may be of importance in clinical practice, to intervene at an early stage which might benefit prognosis in advanced cancer disease.

## Figures and Tables

**Table 1:** Characteristics of the study population.

		All (n = 99) Mean ± SD	Men (n = 55) Mean ± SD	Women (n = 44) Mean ± SD
Age, years		63.9 ± 11.2	65.9 ± 9.4	61.5 ± 12.7
Height, cm		172.3 ± 8.9	176.9 ± 6.6	166.6 ± 8.1
Weight_,_ kg				
	Pre-diagnosis	78.1 ± 13.8	82.8 ± 12.7	72.3 ± 13.0
	Present	71.4 ± 12.1	75.4 ± 10.4	66.2 ± 12.3
Body Mass Index (BMI), kg/m^2^				
	Pre-diagnosis	26.2 ± 3.9	26.4 ± 3.7	26.1 ± 4.3
	Present	24.0 ± 3.7	24.1 ± 3.3	23.9 ± 4.1
		n (%)	n (%)	n (%)
Education, years				
	≤9	12 (12.2)	9 (16.4)	3 (6.8)
	10–12	48 (48.5)	23 (41.8)	25 (56.8)
	>12	39 (39.4)	23 (41.8)	16 (36.4)
BMI defined as overweight/obese prior diagnosis (>25.0 kg/m^2^)		58 (60.4)	32 (60.4)	26 (60.5)
Ever attempted weight loss prior diagnosis		33 (34.7)	12 (23.1)	21 (48.8)
Time since diagnosis, years				
	<1	39 (39.4)	24 (43.6)	15 (34.1)
	1–2	32 (32.3)	16 (29.1)	16 (36.4)
	≥3	28 (28.3)	15 (27.3)	13 (25.9)
		Mean ± SD	Mean ± SD	Mean ± SD
Health-related quality of life (HRQoL)				
	Physical functioning	55.6 ± 26.1	56.4 ± 26.4	54.6 ± 26.0
	Role limitations due to physical health	18.6 ± 32.7	20.4 ± 34.5	16.5 ± 30.9
	Role limitations due to emotional problems	38.7 ± 42.9	45.6 ± 42.9	31.1 ± 42.2
	Energy/fatigue	42.9 ± 22.8	44.8 ± 23.6	40.8 ± 21.8
	Emotional well-being	63.5 ± 20.2	66.7 ± 21.7	59.9 ± 17.9
	Social functioning	51.5 ± 29.8	55.0 ± 30.7	47.4 ± 28.5
	Pain	54.3 ± 30.0	55.4 ± 32.5	53.0 ± 27.1
	General health	40.7 ± 17.1	39.7 ± 17.6	42.0 ± 16.7
Body image score		4.61 ± 4.98	3.61 ± 3.72	5.89 ± 6.05

**Table 2:** Cancer diagnoses in the study population.

	All (n = 99) Men ± SD	Men (n = 55) Mean ± SD	Women (n = 44) Mean ± SD
Breast	3 (3.1)	0 (0.0)	3 (6.8)
Gastrointestinal	23 (23.5)	16 (29.6)	7 (15.9)
Gynecological	7 (7.1)	0 (0.0)	7 (15.9)
Head/neck	13 (13.3)	9 (16.7)	4 (9.1)
Liver	13 (13.3)	10 (18.5)	3 (6.8)
Lung	12 (12.2)	6 (13.6)	6 (13.6)
Pancreas	16 (16.3)	9 (16.7)	7 (15.9)
Prostate	5 (5.1)	5 (9.3)	0 (0.0)
Other	22 (22.5)	12 (22.2)	10 (22.7)

**Table 3:** Linear regression models illustrating associations between weight loss percent and health-related quality of life (HRQoL) and body image distress in those cancer patients that had lost weight (n = 80).

		Men (n = 46)		Women (n = 34)	
		Crude	Age-Adjusted	Crude	Age-Adjusted
		β (95 % CI)	β (95 % CI)	β (95 % CI)	β (95 % CI)
Health-related quality of life (HRQoL)					
	Physical functioning	−1.42 (−3.49, −0.34)	−1.34 (−2.44, −0.24)	0.00 (−1.48, 1.48)	0.03 (−1.45, 1.51)
	Role limitations due to physical health	0.75 (−0.65, 2.14)	0.61 (−0.79, 2.00)	2.00 (0.60, 3.38)	2.02 (0.63, 3.41)
	Role limitations due to emotional problems	−0.10 (−2.11, 1.91)	−0.28 (−2.30, 1.74)	−0.26 (−2.54, 2.01)	−0.33 (−2.57, 1.92)
	Energy/fatigue	−0.27 (−1.39, 0.84)	−0.34 (−1.48, 0.80)	0.47 (−0.63, 1.57)	0.48 (−0.63, 1.59)
	Emotional well-being	0.33 (−0.61, 1.26)	0.20 (−0.73, 1.13)	0.24 (−0.80, 1.27)	0.24 (−0.81, 1.29)
	Social functioning	0.42 (−1.99, 1.82)	0.32 (−1.12, 1.75)	0.60 (−0.84, 2.02)	0.57 (−0.87, 2.01)
	Pain	0.18 (−1.27, 1.62)	0.10 (−1.38, 1.58)	0.90 (−0.57, 2.37)	0.89 (−0.60, 2.38)
	General health	−0.14 (−0.98, 0.70)	−0.05 (−0.90, 0.80)	0.31 (−0.51, 1.13)	−0.32 (−0.52, 1.15)
Body Image distress		0.20 (0.04, 0.36)	0.22 (0.07, 0.37)	0.04 (−0.31, 0.39)	0.04 (−0.31, 0.39)

## Data Availability

Requests for anonymized data not published within this article should be addressed to the principal investigator of the study (YTL). Data is stored at Karolinska Institutet and will be made available by request from any qualified investigator.
